# The Role of High-Content Complex Dietary Fiber in Medical Nutrition Therapy for Gestational Diabetes Mellitus

**DOI:** 10.3389/fphar.2021.684898

**Published:** 2021-07-01

**Authors:** Hong-Kun Wang, De-Cui Cheng, Yue-Min Yang, Xia-Hong Wang, Yan Chen, Lin Zhang, Lian Xiu, Xian-Ming Xu

**Affiliations:** ^1^ Department of Obstetrics and Gynecology, Shanghai General Hospital, Shanghai Jiao Tong University School of Medicine, Shanghai, China; ^2^ Shanghai Jiading Maternal Child Health Hospital, Shanghai, China; ^3^ Shanghai Puto District Maternity and Child Care Hospital, Shanghai, China

**Keywords:** gestational diabetes mellitus, ricnoat, high-content complex dietary fiber, satiety, stools

## Abstract

**Objectives:** A controlled open clinical study was conducted to evaluate the role of Ricnoat, a high-content complex dietary fiber powder produced by Zhuhai Aimed Biotechnology Co. Ltd., in medical nutrition therapy (MNT) to treat gestational diabetes mellitus (GDM). The study aimed to investigate glycemic control, lipid control, weight control, and pregnancy outcomes (neonatal weight) in patients with GDM, as well as evaluate the clinical safety of Ricnoat.

**Methods:** A total of 120 patients with GDM who were admitted to three hospitals in Shanghai between January 2019 and January 2020 were enrolled. Ricnoat was used for intervention for patients in the experimental group. Using a χ^2^ test and *t*-test, respectively, comparisons were conducted between the measurement data and countable data of the demographics and baseline disease characteristics of the experimental group and control group.

**Results:** Fasting blood glucose, 2-h postprandial blood glucose, glycated hemoglobin, total cholesterol, triglycerides, low-density lipoprotein, maternal gestational weight gain, neonatal weight, serum creatinine, glutamate transaminase, and aspartate aminotransferase were lower in the experimental group than in the control group, whereas high-density lipoprotein was higher in the experimental group than in the control group. Ricnoat intervention resulted in satiety higher than the expected 80% and more common occurrence of type 4 (smooth and soft, like salami or a snake) and type 5 (a soft mass with clear edges) stools.

**Conclusion:** Ricnoat intervention had a significant effect on glycemic control, lipid control, weight control, and pregnancy outcomes (neonatal weight) in patients with GDM by enhancing maternal satiety and improving the stool features of pregnant women. It was also found to be safe for application during pregnancy.

## Introduction

Current research shows that, in China, gestational diabetes mellitus and abnormal glucose metabolism in pregnancy occur in 17% of pregnant women (Zhu et al., 2015). This is accompanied by the increased incidence of excessive and rapid GWG, cesarean delivery, macrosomia, and neonatal hypoglycemia. It has also been found that lactating women are experiencing more prolonged postpartum weight retention and greater difficulty with breastfeeding (Zhang et al., 2008).

With the application of medical nutrition therapy, 90% of pregnant women with GDM can effectively control their blood glucose, ensuring normal fetal development, an appropriate fetal birth weight, and a good delivery outcome (Zhang et al., 2016). However, a large number of patients with GDM who receive MNT experience significant hunger, especially during late pregnancy, resulting in increased food and caloric intake, which in turn leads to the failure of MNT (Dolatkhah et al., 2018). The present study increased the content of dietary fiber in the limited caloric intake of pregnant women to evaluate its efficacy in lowering blood glucose while offering a sense of satiety, thereby allowing the reduction of food and caloric intake, stabilizing blood glucose, delaying GWG, and reducing the incidence of macrosomia.

## Materials and Methods

### Subjects

A total of 120 patients with GDM who were admitted to three hospitals in Shanghai between January 2019 and January 2020 were enrolled in this study.

Inclusion criteria: Patients aged 25–35 years (singleton, first pregnancy) with the diagnosis of GMD at 24–28 weeks of gestation.

Exclusion criteria: 1) Pregnant patients with diabetes mellitus; 2) before pregnancy, fasting blood glucose (FBG) ≥ 7.0 mmol/L, 2-h blood glucose ≥11.1 mmol/L in a 75 g oral glucose tolerance test (OGTT); 3) symptoms of diabetes together with random blood glucose ≥11.1 mmol/L (WHO, 2013); 4) patients who were unable to comply with the observations due to drug abuse or psychiatric factors.

Deletion criteria: 1) Patients who did not take the medication according to the study protocol, with a missed-dose rate ≥20%; 2) patients who had no records after medication.

Criteria for discontinuation of the study: 1) Patients who could not continue with the observations due to abdominal distension or other adverse effects; 2) patients who were lost contact during the follow-up.

The diagnosis of GDM followed the International Association of Diabetes and Pregnancy Study Groups criteria; that is, when one or more of the following blood glucose indicators met or exceeded the diagnostic criteria, with the adoption of a 75 g OGTT to screen the patients at 24–28 weeks of gestation (after at least 8 h of fasting): FBG 5.1 mmol/L, 1-h blood glucose 10 mmol/L, 2-h blood glucose 8.5 mmol/L.

## Study Methods

### Ricnoat

High-content complex dietary fiber powder (Ricnoat), 18 g/packet, each packet containing 1.54 g ß-dextran, 10 packets/box. Producer: Zhuhai Aimed Biotechnology Co. Ltd. Shelf life: 24 months. Storage method: Store in a cool, dry, and dark place.

### Therapeutic Protocol

**FIGURE 1:**
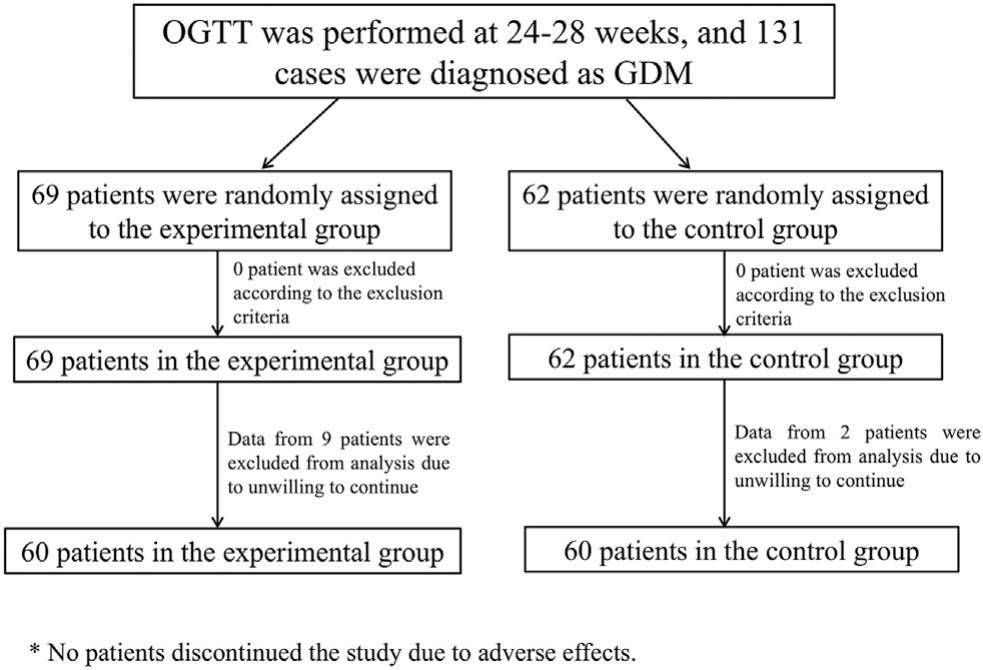
The flow chart of enrollment and grouping.

Patinets were randomly assigned into two groups: the control group (Group A, *n* = 60) and the experimental group (Group B, *n* = 60). Patients in Group A (control group) adjusted their diet and lifestyle according to MNT standard meals and did not use any medications or health foods that might affect blood glucose and lipids. Patients in Group B (experimental group) adjusted their diet and lifestyle according to MNT standard meals, with the addition of 18 g of Ricnoat twice a day, 30 min before breakfast and dinner, for eight consecutive weeks. Dosing method: add 150 ml of boiled water and stir well before administration. Supplement with more than 100 ml of liquid after each dose. There is no combined medication in two group. The flow chart of enrollment and grouping is shown in Figure 1.

### Observation Indicators and Time Points of Detection

Observation indicators: Blood glucose (FBG, 2-h postprandial glucose, and glycated hemoglobin [HbA1c]), blood lipids (total cholesterol [TC], triglycerides [TG], low-density lipoprotein [LDL], and high-density lipoprotein [HDL]), liver and kidney function, body weight, satiety, and defecation (stool features). Postprandial blood glucose measurement started at the beginning of the meal. The data of satiety and defecation (stool features) were obtained by filling in a predesigned questionnaire form (Supplementary Material). The results are shown in Table 1. The pregnancy outcomes of the two groups were followed up: delivery mode, gestational age of delivery, neonatal birth weight, macrosomia, low birth weight, neonatal Apgar score and the incidence of preeclampsia.

**TABLE 1:** The satiety at different stages in the pregnant females with oral administration of Ricnoat in the Ricnoat group.

Score	Grade	Satiety 1 W	Satiety 4 W	Satiety 8 W
1	Hunger	0	0	0
2		0	0	0
3		1	0	1
4	Moderate	14	15	16
5		35	35	38
6	Overfed	3	7	3
7		5	2	1
8		2	1	0
9		0	0	0
10		0	0	1
Total		60	60	60
Moderate satiety proportion		81.67%	83.33%	90.00%

Time points of detection: At enrollment and every week for 8 weeks after the treatment. All centers monitored the biochemical indicators using the kits with the same batch number, and traced blood glucose using the Johnson & Johnson One-Touch microglucose meter.

## Statistical Analysis

Using a χ^2^ test and *t*-test respectively, comparisons were conducted between the measurement data and co untable data of the demographics and baseline disease characteristics of the experimental group and control group. *p* < 0.05 was considered statistically significant.

RESULTS

### General Characteristics

The general characteristics of the enrolled patients are shown in Table 2. Between the two groups, HbA1c in the Ricnoat group was higher than that in the control group at before treatment and 28 days after treatment, and 56 days after treatment. There were no statistical differences in age, OGTT values at various periods, weight and height before pregnancy between the two groups.

**TABLE 2:** The general characteristics of the enrolled pregnant females.

Item	Ricnoat group	Control group	*p*
Age (years)	30.23 ± 3.78	30.47 ± 4.19	0.701
OGTT 0 h	4.95 ± 0.58	5.04 ± 0.54	0.560
OGTT 1 h	10.07 ± 1.5	9.48 ± 2.15	0.670
OGTT 2 h	8.93 ± 1.44	8.28 ± 1.52	0.080
Weight before pregnancy (kg)	59.43 ± 13.93	58.54 ± 8.65	0.620
Height (cm)	160 ± 5.59	162.46 ± 5.46	0.083
HbA1 before treatment	5.06 ± 0.38	4.97 ± 0.4	0.019
HbA1c 28 d	5.09 ± 0.39	4.95 ± 0.34	0.034
HbA1c 56 d	5.21 ± 0.44	5.27 ± 0.35	0.028

### Blood Glucose at Different Time Points

There was no statistical difference between the two groups in the initial FBG. The intervention of dietary fiber, however, caused gradual differences between the two groups over time, with the FBG in the experimental group being lower than that in the control group at every time point after intervention. These differences were statistically significant (*p* < 0.05). HbA1c in pregnant women in the experimental group was slightly higher than that in the control group before intervention, but the increase in the experimental group was significantly lower than that in the control group after intervention. These data indicated that the HbA1c level in pregnant women with GDM increased gradually with the increase in gestational age. However, less increase was observed after the Ricnoat intervention, suggesting that Ricnoat had a positive effect on maintain blood glucose level.

**FIGURE 2:**
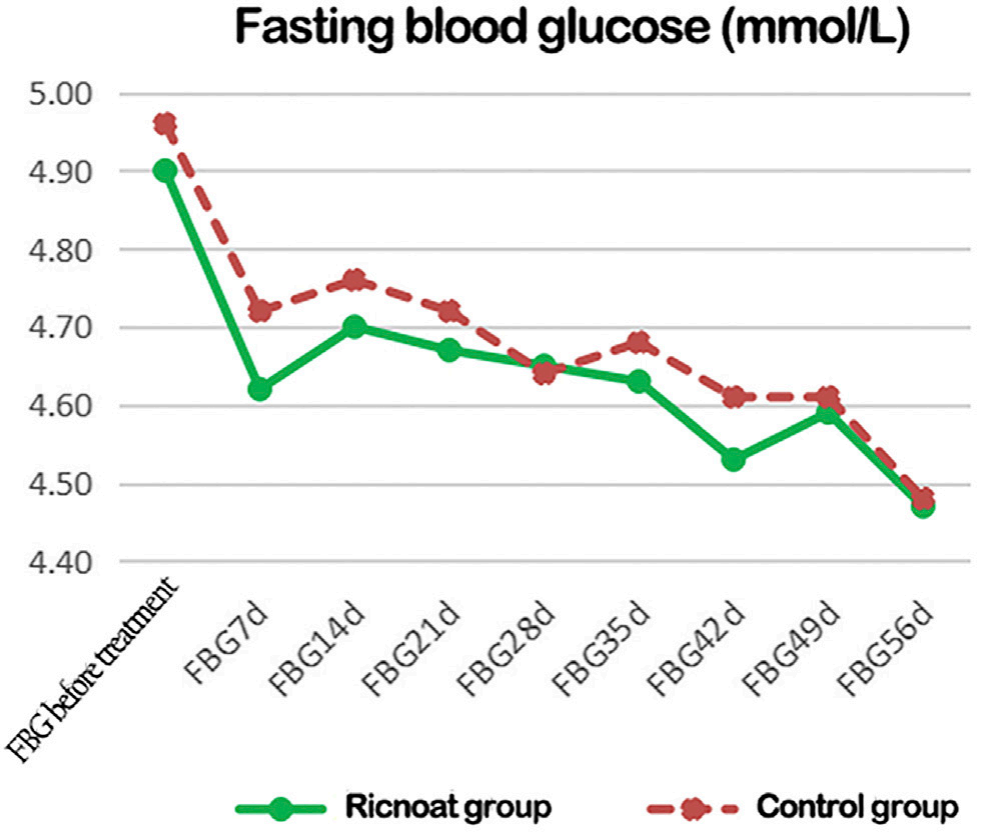
The changes of blood glucose at different time points between the two groups of patients.

The results are shown in Figure 2 and Table 3.

**TABLE 3:** The comparison of the fasting blood glucose at different time points between the two groups of pregnant females.

FBG (mmol/L)	Ricnoat group	Control group	*p*
FBG before treatment	4.901 ± 0.63	4.96 ± 0.61	0.060
FBG 7 d	4.62 ± 0.56	4.72 ± 0.54	0.041
FBG 14 d	4.7 ± 0.56	4.76 ± 0.4	0.032
FBG 21 d	4.67 ± 0.52	4.72 ± 0.49	0.036
FBG 28 d	4.65 ± 0.53	4.64 ± 0.46	0.048
FBG 35 d	4.63 ± 0.39	4.68 ± 0.46	0.037
FBG 42 d	4.63 ± 0.45	5.23 ± 5.37	0.042
FBG 49 d	4.59 ± 0.49	4.61 ± 0.49	0.021
FBG 56 d	4.47 ± 0.5	4.48 ± 0.45	0.039

**FIGURE 3:**
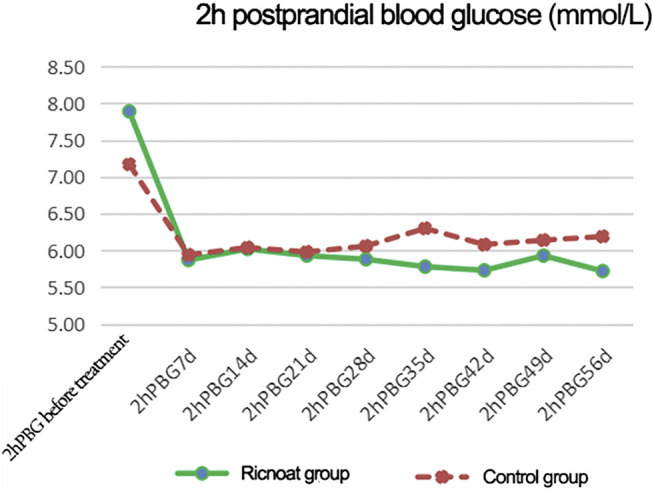
The changes of 2-h postprandial blood glucose at different time points between the two groups of patients.

The initial 2-h postprandial blood glucose in the experimental group was slightly higher than that in the control group. The intervention of dietary fiber caused gradual differences between the two groups over time, and the 2-h postprandial blood glucose in the experimental group was lower than that in the control group at every time point after intervention. These differences were statistically significant (*p* < 0.05). The results are shown in Figure 3and Table 4.

**TABLE 4:** The comparison of the 2-h postprandial blood glucose at different stages between the two groups of pregnant females.

2hPBG (mmol/L)	Ricnoat group	Control group	*p*
2hPBG before treatment	7.89 ± 1.81	7.17 ± 1.58	0.043
2hPBG 7 d	5.87 ± 0.86	5.94 ± 1.09	0.032
2hPBG 14 d	6.02 ± 0.88	6.04 ± 0.95	0.047
2hPBG 21 d	5.93 ± 0.77	5.98 ± 1.35	0.018
2hPBG 28 d	5.88 ± 0.91	6.06 ± 1.05	0.020
2hPBG 35 d	5.78 ± 0.7	6.3 ± 1.27	0.028
2hPBG 42 d	5.73 ± 0.75	6.08 ± 1.09	0.048
2hPBG 49 d	5.93 ± 0.99	6.14 ± 0.84	0.029
2hPBG 56 d	5.72 ± 0.83	6.19 ± 0.93	0.043

### Blood Lipids at Different Time Points

Before intervention, there was a statistically significant difference in TC levels between the two groups. At week 8 after intervention, the TC levels in the experimental group were 6.08 ± 1.21 mmol/L, whereas in the control group they were 6.49 ± 1.29 mmol/L; the difference was statistically significant.

The TG levels in the experimental group before intervention were 2.01 ± 0.74 mmol/L, while in the control group they were 1.92 ± 0.65 mmol/L; the difference was statistically significant. At week 8 after intervention, the TG levels in the experimental group were 3.05 ± 1.01 mmol/L, this being lower than the TG levels in the control group (3.23 ± 1.47 mmol/L); the difference was statistically significant.

At most of the time points, there were no statistically significant differences in HDL levels between the two groups. However, at week 4 and week 8 after intervention, the HDL levels in the experimental group were 1.83 ± 0.41 mmol/L and 1.79 ± 0.49 mmol/L, respectively, these being higher than the HDL levels in the control group (1.75 ± 0.38 mmol/L and 1.75 ± 0.49 mmol/L, respectively); the differences were statistically significant.

The initial LDL levels in the experimental group were slightly higher than those in the control groupwhich were 2.87 ± 0.79 and 2.86 ± 0.9 mmol/L, respectively. At week 4 and week 8 after intervention, however, the LDL levels of the experimental group were lower than those of the control group which were 3.15 ± 0.88, 3.32 ± 0.89 mmol/L and 3.08 ± 0.89, 3.48 ± 1.01 mmol/L, respectively. And the differences were statistically significant. The blood lipid results are shown in Table 5.

**TABLE 5:** The comparison of the blood lipids at different stages between the two groups of pregnant females.

Blood lipids type (mmol/L)	Ricnoat group	Control group	*p*
TC before treatment	5.43 ± 1.15	5.59 ± 1.22	0.034
TC 28 d	6.02 ± 1.16	6.34 ± 1.19	0.010
TC 56 d	6.08 ± 1.21	6.49 ± 1.29	0.003
TG before treatment	2.01 ± 0.74	1.92 ± 0.65	0.036
TG 28 d	2.8 ± 1.37	2.87 ± 1.09	0.102
TG 56 d	3.05 ± 1.01	3.23 ± 1.47	0.045
HDL-C before treatment	1.83 ± 0.41	1.80 ± 0.40	0.088
HDL-C 28 d	1.83 ± 0.41	1.75 ± 0.38	0.039
HDL-C 56 d	1.79 ± 0.49	1.75 ± 0.49	0.049
LDL-C before treatment	2.87 ± 0.79	2.86 ± 0.9	0.027
LDL-C 28 d	3.15 ± 0.88	3.32 ± 0.89	0.031
LDL-C 56 d	3.08 ± 0.89	3.48 ± 1.01	0.043

### Maternal Weight Control During Pregnancy and Neonatal Outcomes

There were no statistically significant differences in body weight and body mass index (BMI) between the two groups before intervention. At week 4 and week 8 after intervention, however, bodyweight (66.84 ± 8.74 kg and 68.79 ± 12.8 kg, respectively) and BMI (25.29 ± 3.07 kg/m^2^ and 26.16 ± 3.09 kg/m^2^, respectively) in the experimental group were lower than the corresponding body weight (67.11 ± 12.71 kg and 69.11 ± 9.18 kg, respectively) and BMI (26.24 ± 4.33 kg/m^2^ and 26.89 ± 4.38 kg/m^2^, respectively) in the control group, and the differences were statistically significant. In addition, there were no significant differences in delivery mode, gestational age of delivery, macrosomia, low birth weight, Apgar score of neonates and the incidence of preeclampsia. The birth weight of neonates in the experimental group was lower than that in the control group. The bodyweight of the neonates in the experimental group was 3,197.42 ± 417.15 g, while in the control group it was 3,346.67 ± 330.69 g. And the differences were statistically significant (Table 6).

**TABLE 6:** The comparison of the weight control and the outcome of the neonates between the two groups of pregnant females.

Weight control	Ricnoat group	Control group	*p*
Weight before treatment (kg)	65.11 ± 13.78	64.43 ± 8.48	0.056
Weight 28 d	66.84 ± 8.74	67.11 ± 12.71	0.023
Weight 56 d	68.79 ± 12.8	69.11 ± 9.18	0.036
BMI before treatment (kg/m^2^)	25.43 ± 4.75	24.4 ± 3.02	0.071
BMI 28 d	25.29 ± 3.07	26.24 ± 4.33	0.047
BMI 56 d	26.16 ± 3.09	26.89 ± 4.38	0.029
Newborn weight (g)	3,197.42 ± 417.15	3,346.67 ± 330.69	0.030

### Liver and Renal Function

Before intervention, there was no statistically significant difference in AST levels between the two groups. At week 8 after intervention, however, it was found that the AST levels in the experimental group (14.53 ± 4.4 U/L) were lower than those in the control group (18.83 ± 10.39 U/L), although both were within the normal reference range and the differences were statistically significant.

Before intervention, the ALT were 17.18 ± 7.59 and 21.14 ± 12.51 μmmol/L respectively, and SCr were 40.95 ± 6.96 and 44.56 ± 7.61 μmmol/L respectively, levels of the experimental group were lower than those of the control group. At week 8 after intervention, these levels were still lower in the experimental group than those in the control group. The differences were statistically significant, but the levels in both groups were within the normal reference range (see Table 7).

**TABLE 7:** The comparison of the hepatic and renal function between the two groups of pregnant females.

Hepatic and renal function (μmol/L)	Ricnoat group	Control group	*p*
AST before treatment	16.8 ± 6.85	19.16 ± 8.46	0.096
AST 56 d	14.53 ± 4.41	18.83 ± 10.39	0.004
ALT before treatment	17.18 ± 7.59	21.14 ± 12.51	0.038
ALT 56 d	13.31 ± 5.82	15.76 ± 8.32	0.045
SCr before treatment	40.95 ± 6.96	44.56 ± 7.61	0.008
SCr 56 d	47.69 ± 10.83	48.02 ± 9.93	0.013

### Satiety and Defecation in the Experimental Group

The results of the questionnaire survey in the experimental group suggested that the patients experienced moderate satiety at weeks 1, 2, and 3 after intervention (81.67, 83.33, and 90.00%, respectively), and these results were higher than the expected outcome of 80%.

After oral administration of Ricnoat, the stool features of the patients in the experimental group at 28 days after treatment and 56 days after treatment were investigated, with type 4 (smooth and soft, like salami or a snake) and type 5 (a soft mass with clear edges) stools being the most common (see Tables 1, 8). Also, the number of a fecal traits score ≤3 in the Ricnoat group was significantly less than that in the control group, suggesting that Ricnoat had an effect on reducing constipation and improving fecal traits.

**TABLE 8:** The defecation (stool features) of pregnant females in the Ricnoat and Control groups at different stages.

Stool type	14 d		21 d		28 d		35 d		42 d		49 d		56 d	
	Recnoat group	Control group	Ricnoat group	Control group	Ricnoat group	Control group	Ricnoat group	Control group	Ricnoat group	Control group	Ricnoat group	Control group	Ricnoat group	Control group
1	1	2	1	2	1	2	0	3	0	3	0	3	1	3
2	5	7	1	8	2	8	2	9	3	8	3	8	1	9
3	2	3	2	3	7	3	7	2	8	4	6	4	8	5
4	29	30	36	32	32	31	35	32	33	30	27	28	32	28
5	22	18	19	14	16	14	14	12	15	13	20	14	16	12
6	1	0	1	1	2	2	2	2	1	2	4	3	2	3
7	0	0	0	0	0	0	0	0	0	0	0	0	0	0
Total	60	60	60	60	60	60	60	60	60	60	60	60	60	60

## Discussion

Dietary fiber is one of the seven major types of nutrients. It is closely correlated with human health, and increasing it in a diet is a simple and effective means of dietary control in the treatment of GDM. Dietary fiber comes in two forms-insoluble cellulose and soluble cellulose, both of which play a role in water absorption, swelling, and wrapping and are substrates for the growth and fermentation of probiotics. Insoluble fiber has a key role in promoting intestinal peristalsis, while soluble fiber is important in adhesion and can slow down the absorption of glucose (Fang and Liu, 2015).

Previous studies have confirmed that dietary fiber is one of the most important factors in maintaining normal insulin sensitivity in human tissue cells (Cameron-Smith et al., 1997; Weickert et al., 2006; Rave et al., 2007), and inadequate intake can lead to a high incidence of diabetes mellitus. (Salmeron et al., 1997; Jiang et al., 2012; Post et al., 2012; Wang, Wang)Studies have also shown that increasing the intake of dietary fiber can reduce the FBG and HbA1C levels in diabetic patients (Zhang et al., 2008; WHO, 2013; Fang and Liu, 2015), increase insulin sensitivity, improve existing insulin resistance, and reduce the impacts of diabetes upon the patient (Liese et al., 2005; Zhang and Zhang, 2008; Fang et al., 2009; Robertson et al., 2012). One study concluded that, based on drug therapy, long-term supplementation with an adequate amount of dietary fiber can restore the insulin sensitivity in some patients with type 2 diabetes to the normal levels, thus correcting the glucose metabolism (Liu et al., 2016).

Oats contain the soluble dietary fiber ß-dextran. Some studies have shown that soluble oat fiber can reduce the risk of GDM by 26% (Othman et al., 2011). Other studies have suggested that an intake of 5–10 g/day of soluble dietary fiber can reduce serum cholesterol by 5–10%, while an increase of 10 g/day of dietary fiber (or 10 g/day of insoluble dietary fiber from cereals) can reduce the risk of coronary heart disease and type 2 diabetes by 30% (Shen et al., 2016).

Ricnoat, a high-content complex dietary fiber powder, has been specially developed for patients with diabetes and dyslipidemia and patients who are overweight, so it is suitable for MNT in patients with metabolic diseases. Each packet of Ricnoat contains up to 9.5 g of dietary fiber, so one or two packets a day can effectively supplement a lack of dietary fiber. The soluble dietary fiber and insoluble dietary fiber in Ricnoat are proportioned at a ratio of 6:4, which more closely addresses the dietary needs of patients who are overweight and those with abnormal glucose and lipid metabolism.

Ricnoat consists of dietary fiber from oat, wheat, soy, and corn, so it is more comprehensive than a single dietary fiber product and meets the human body’s need for different dietary fibers. It is therefore in line with the principles of food diversity and the cereal-based dietary intake recommended in the Chinese Dietary Guidelines of 2016.

The 2015 guidelines for the MNT of diabetes in China clearly state that the dietary fiber intake of patients with diabetes should meet and exceed the recommended intake for patients without diabetes (25∼30 g/day). One study has shown that the dietary fiber intake of pregnant women with abnormal glucose metabolism in China is only 60% of the normal requirement (Shen et al., 2016). Other studies have shown that dietary fiber can improve blood glucose and lipids in patients with diabetes(Rivellese et al., 1980; Riccardi et al., 1984). One prospective cohort study demonstrated that, in pregnant women with GDM, intervention with a high-content complex dietary fiber significantly improved blood glucose levels (Reynolds et al., 2020), including FBG, 2-h postprandial blood glucose, and HbA1C. Dietary fiber can delay and reduce the absorption of carbohydrates in the small intestine, allowing the body to fully secrete insulin. It can also ferment in the large intestine to produce short-chain fatty acids, which improve the balance of intestinal flora and increase the secretion of insulin (Lattimer and Haub, 2010; Tolhurst et al., 2012).

A higher intake of total and insoluble dietary fiber has been found to reduce the risk of diabetes by lowering the levels of inflammatory markers (such as fibrinogen activator inhibitor-1, resistin, C-reactive protein, and interleukin-6) (Wannamethee et al., 2009). Soluble dietary fiber alone improves glucose and insulin responses by delaying gastric emptying and nutrient absorption and can suppress postprandial glucose elevation in patients with limited insulin secretion (Fujimoto, 1996).

In the present study, the differences in lipid indicators (TG, TC, LDL, and HDL) between the experimental group and the control group were statistically significant, indicating that supplementation with a high-content complex dietary fiber could improve blood lipid levels in pregnant women (Jovanovski et al., 2019). It was also found that the patients in the experimental group had lower levels of GWG than the control group, which was consistent with the findings of a previous study (Tucker and Thomas, 2009).

The results of the present study suggest that Ricnoat can enhance the satiety of pregnant women, which might be correlated with three factors: 1) soluble fiber is fermented in the large intestine to produce glucagon-like peptide and gastrointestinal hormone peptide YY (McRae et al., 2006), two intestinal hormones that can play a role in the induction of satiety; 2) dietary fiber significantly reduces energy intake, so a greater consumption of fiber may lead to a decreased need to consume dietary fat (Unnikrishnan et al., 2017); 3) dietary fiber reduces dietary metabolic energy, which is total energy minus energy lost in feces, urine, and combustible gases.

An increase in dietary fiber leads to a decrease in fat digestibility, meaning simple carbohydrate intake might decrease. Although dietary fiber is still part of the total calories in the diet, it is highly resistant to digestion in both the small intestine and the large intestine, so it can lubricate the stools. This could explain the softer and smoother stool features of the patients in the experimental group in the present study.

The present study had some limitations, and it only evaluated the presence or absence of dietary fiber in pregnant patients with GDM. Further research is required to determine the timing and amount of dietary fiber supplements. The sample size of this study is still not large, and the sample size can be expanded in the future to further explore the role of dietary fiber in GDM management. Dietary fiber can also be used to prevent GDM in high-risk groups.

## Conclusion

A high-fiber diet is an important part of managing GDM (Reynolds et al., 2019). It can improve blood glucose and blood lipids, help patients maintain a healthy bodyweight. Avoiding excessive birth weight may have a role in preventing macrosomia. Supplementation with Ricnoat effectively improves the satiety and stool features of pregnant women with GDM. It is also safe for application during pregnancy.

## Data Availability

The original contributions presented in the study are included in the article/Supplementary Material, further inquiries can be directed to the corresponding author.
