# Corrigendum: The Role of High-Content Complex Dietary Fiber in Medical Nutrition Therapy for Gestational Diabetes Mellitus

**DOI:** 10.3389/fphar.2021.757887

**Published:** 2021-09-09

**Authors:** Hong-Kun Wang, De-Cui Cheng, Yue-Min Yang, Xia-Hong Wang, Yan Chen, Lin Zhang, Lian Xiu, Xian-Ming Xu

**Affiliations:** ^1^ Department of Obstetrics and Gynecology, Shanghai General Hospital, Shanghai Jiao Tong University School of Medicine, Shanghai, China; ^2^ Shanghai Jiading Maternal Child Health Hospital, Shanghai, China; ^3^ Shanghai Putuo District Maternal and Infant Health Care Hospital, Shanghai, China

**Keywords:** gestational diabetes mellitus, ricnoat, high-content complex dietary fiber, satiety, stools

In the published article, there was an error in the list of author affiliations. Instead of “**1 Department of Obstetrics and Gynecology, Jiao Tong University School of Medicine, Shanghai, China**” and “**4 Shanghai General Hospital Shanghai, Shanghai, China**”, affiliation 1 should have been listed as “**1. Department of Obstetrics and Gynecology, Shanghai General Hospital, Shanghai Jiao Tong University School of Medicine**”. Affiliation 4 has subsequently been removed, and author Xian-Ming Xu’s affiliation changed from 4 to 1.

The authors apologize for this error and state that this does not change the scientific conclusions of the article in any way. The original article has been updated.

