# Supplementary material for: The Role of High-Content Complex Dietary Fiber in Medical Nutrition Therapy for Gestational Diabetes Mellitus
Source: Front Pharmacol. 2021 Jul 1;12:684898. doi: 10.3389/fphar.2021.684898 (PMC8281130; doi:10.3389/fphar.2021.684898)
Supplement: Supplementary file 1 [file Datasheet1.DOCX]

Appendix（1）Satiety score after administration of Ricnoat (self-test)

| Grade | Symptom description | Score | Time | | |
| --- | --- | --- | --- | --- | --- |
|  |  |  | The first week | The fourth week | The eighth week |
| Overfed | Stuffed stomach with a feeling of nausea | 10 | □ | □ | □ |
|  | Uncomfortably full, need to relax the belt | 9 | □ | □ | □ |
|  | Uncomfortably full，feel can't eat anymore | 8 | □ | □ | □ |
|  | Uncomfortably full，fell overfed | 7 | ☑ | ☑ | □ |
|  | Comfortably full with satisfaction、 | 6 | □ | □ | □ |
| Proper | Proper, neither hungry nor overfed | 5 | □ | □ | □ |
|  | A bit hunger | 4 | □ | □ | ☑ |
| Hunger | Hunger, want to eat, and endurable | 3 | □ | □ | □ |
|  | Very hungry and cannot concentrate on something | 2 | □ | □ | □ |
|  | Dizzy with hunger and a rumbling stomach | 1 | □ | □ | □ |

Appendix（2）The record of stool features with the administration of Ricnoat

| Time | Stool features | | | | | | |
| --- | --- | --- | --- | --- | --- | --- | --- |
| 7d | 1 | 2 | 3 | 4 | 5 | 6 | 7 |
| 14d | □ | □ | □ | □ | □ | □ | □ |
| 21d | □ | □ | □ | □ | □ | □ | □ |
| 28d | □ | □ | □ | □ | □ | □ | □ |
| 35d | □ | □ | □ | □ | □ | □ | □ |
| 42d | □ | □ | □ | □ | □ | □ | □ |
| 49d | □ | □ | □ | □ | □ | □ | □ |
| 56d | □ | □ | □ | □ | □ | □ | □ |

Appendix（3）Bristol stool typing chart

| Stool types | Description of the stool features | Stool types | Illustration of stool features | Description of the stool features | Illustration of stool features |
| --- | --- | --- | --- | --- | --- |
| 1 | Scattered hard pieces, resembling nuts | 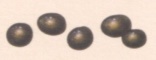 | 2 | Sausage-like, but in chunks | 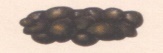 |
| 3 | Sausage-like, with cracks on the surface | 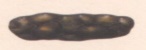 | 4 | Resembles salami or snake, smooth and soft | 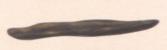 |
| 5 | Soft mass with clear edges | 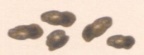 | 6 | Fluffy material, unclear edges, paste-like stool | 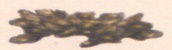 |
| 7 | Watery stool, with no solids | 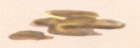 |  | | |
